# Prevalence of Gastroesophageal Reflux Disease and Its Impact on the Quality of Life Among Obese Individuals in Al-Baha Region, Saudi Arabia

**DOI:** 10.7759/cureus.63073

**Published:** 2024-06-24

**Authors:** Ahmed T Elshennawy, Ibrahim M Shatla, Ramy H Agwa, Hind A Alghamdi, Saud Turki N Alghamdi, Abdullah Mohammad M Alnashri, Sarah Dhaifallah S Alqarni, Sami Saeed B Alghamdi, Salwa Ibrahim M Alghamdi, Mohammed Abdulhadi M Alghamdi

**Affiliations:** 1 Department of Anatomy, Faculty of Medicine, Al-Baha University, Al-Baha, SAU; 2 Department of Physiology, Faculty of Medicine, Al-Baha University, Al-Baha, SAU; 3 Department of Physiology, Faculty of Medicine, Al-Azhar University, Damietta, EGY; 4 Department of Internal Medicine/Hepatology and Gastroenterology, Mansoura University, Mansoura, EGY; 5 Department of Internal Medicine/Hepatology and Gastroenterology, Faculty of Medicine, Al-Baha University, Al-Baha, SAU; 6 Faculty of Medicine, Al-Baha University, Al-Baha, SAU

**Keywords:** al-baha, gastroesophageal reflux disease, obese, prevalence, quality of life

## Abstract

Background

Gastroesophageal reflux disease (GERD) is a global gastrointestinal disorder, and obesity is a particular risk factor. Symptoms of GERD, such as heartburn and acid reflux, are caused by abnormal relaxation in the lower esophagus, causing gastric acid reflux. Persistent symptoms can affect the patient’s quality of life (QOL) and can cause complications, such as esophageal adenocarcinoma. Management of GERD includes lifestyle changes, antacids, and anti-reflux surgery. Even though GERD is a common disease, few research has been carried out on it in Saudi Arabia.

Aim

This study aimed to estimate the prevalence of GERD and its associated risk factors among obese individuals in the Al-Baha region population and the effect of GERD on their QOL.

Methods

A cross-sectional study included 314 obese participants from the Al-Baha region. A questionnaire was filled out to measure the prevalence of GERD, risk factors, and effects on the QOL of the participants. Data were analyzed by the IBM SPSS Statistics for Windows, version 26.0 (released 2019, IBM Corp., Armonk, NY). Descriptive statistics and the chi-squared test were applied. Logistic regression analysis was used to determine the factors associated with the incidence of GERD. A *p*-value of <0.05 was considered statistically significant.

Results

A total of 314 patients who met our inclusion criteria completed the survey; 42% of them were women, the mean age of all patients was 35.3 ± 12.9 years, and 38.2% of the patients were diagnosed with GERD. Epigastric pain and burning sensation were the most common symptoms (44.9%). Five out of six domains in the QOL questionnaire showed more effects among GERD participants than non-GERD participants, and the results were statistically significant (*p* = 0.001). Logistic regression analysis showed that men are 1.8 times more likely than women to be diagnosed with GERD, and smokers have 2.6 times the risk of being diagnosed with GERD than non-smokers.

Conclusion

The present study showed a high prevalence of GERD among obese patients in the Al-Baha region, negatively affecting their QOL. Major risk factors included gender, smoking, dyslipidemia, and hypertension. Public health programs to raise awareness of these risk factors and lifestyle habits are necessary to improve QOL and prevent complications.

## Introduction

Gastroesophageal reflux disease (GERD) is a worldwide gastrointestinal disease and represents the most prevalent disease requiring medical advice from a gastroenterologist [1]. The condition occurs when the patient experiences heartburn, acid regurgitation, and chest and epigastric discomfort due to abnormal relaxation in the lower esophageal sphincter (LES), leading to reflux of gastric acid into the lower esophagus [2].

Multiple risk factors have been associated with the development of GERD; fortunately, many of them are modifiable risk factors, such as obesity, smoking, consumption of spicy food, alcohol, and nonsteroidal anti-inflammatory drugs [3-5]. Obesity represents a major risk factor for the development of GERD and associated complications of longstanding untreated reflux, such as adenocarcinoma and Barrett’s esophagus. The pathophysiology depends on the obesity characteristics; in particular, central obesity increases the pressure inside the abdomen, lowers the pressure in the esophageal sphincter, and causes disturbance in gastric emptying, all of which could lead to increased acid reflux [6].

Recurrent and persistent symptoms of GERD affect the quality of life (QOL) and physical life of patients, affecting their daily activities. GERD also affects the healthcare system when the disease progresses and complications, such as esophageal adenocarcinoma, develop [7].

The prevalence of GERD worldwide ranges from 10% to 25%, but the prevalence in Saudi Arabia widely ranges from 20% to 61%. Almadi et al. reported that 45.4% of Riyadh residents have GERD [8], Alsulobi et al. found that 61.8% of residents of Arar City have GERD [9], and Al Ghadeer et al. (2021) reported that 20.4% of the population of the Eastern Province of Saudi Arabia have GERD [10]. Epidemiological data lately have proven a rising GERD prevalence worldwide [11].

Management of GERD is divided into lifestyle modification, antacid medication, and anti-reflux surgery. Lifestyle modifications are recommended for all GERD patients, but absolute dietary restrictions are of limited benefit. Weight gain exacerbates the symptoms of the condition, and even modest weight loss reduces these symptoms. Nocturnal lifestyle modifications, such as raising the head of the bed, are important. Quitting smoking and alcohol helps to reduce GERD symptoms, as well as having many other health benefits [12].

Proton pump inhibitors (PPIs) are the mainstay in the management of GERD, causing refluxate adjustment in its pH to be mildly acidic or alkaline. It should be taken 30 to 45 minutes before eating. Long-term PPI therapy is recommended for proven GERD [13]. It alleviates specific GERD symptoms, such as esophagitis, in 72-83% of patients and resolves heartburn in 56-77% of patients with esophagitis [14].

By blocking brief relaxations of the LES, gamma-aminobutyric acid type B agonists, such as baclofen, reduce acid reflux and may also reduce non-acid reflux [15]. Sucralfate, a complex of sucrose sulfate and aluminum hydroxide, also provides rapid relief from GERD attacks [16]. Prokinetic agents considered for GERD symptoms may reduce symptoms when combined with PPI therapy [17].

Anti-reflux surgery is the final stage of management for resistant or prolonged GERD symptoms or severe structural disturbance at the esophagogastric junction. Anti-reflux surgery, also known as fundoplication, is a procedure aimed at controlling and reinforcing the LES, the muscle that connects the esophagus and stomach and prevents stomach acid from flowing back into the esophagus, but it becomes weak in GERD. Surgery involves wrapping the upper stomach around the LES to stabilize it, preventing acid reflux, and repairing the hiatal hernia, as this can significantly reduce the recurrence of heartburn and GERD symptoms [18,19].

However, although it is a very common gastrointestinal disease, few studies have been published about GERD or its effect on the QOL in Saudi Arabia. No current study has assessed the prevalence of GERD among obese persons in the Al-Baha region. Therefore, this study aims to estimate the prevalence of GERD among obese persons and risk factors among the Al-Baha region population and the effect of GERD on their QOL. It introduces data about GERD and associated risk factors to decrease the disease burden on individuals and the healthcare system.

## Materials and methods

A cross-sectional study was carried out among the population of the Al-Baha region in Saudi Arabia to assess the prevalence of GERD among obese people and its associated risk factors.

To determine people of the Al-Baha region who were obese, the present study classified them according to the body mass index (BMI), where a value of ≥30 kg/m^2^ was considered obese. A random sample of residents of the Al-Baha region was included, using a recognized sample size calculation to determine the sample size.

Inclusion criteria

All participants lived in the Al-Baha region, Saudi Arabia, had a BMI ≥30 kg/m^2^, and agreed to participate in the study.

Exclusion criteria

The study excluded any participants with a BMI <30 kg/m^2^, who did not live in the Al-Baha region, or who did not agree to participate in the survey study. In addition, pregnant women were excluded.

Ethical consideration

The study was conducted after obtaining ethical approval from the Institutional Research Board of Al-Baha University (number REC/MED/BU-FM/2023/91). The participants were informed about the study aims and assured of data confidentiality, and consent was obtained from each participant before participating in the study.

Sampling procedure

The study included members of the general population who met the inclusion criteria. The sample size was calculated using the established formula n = Z^2^ × P(1 − P) / d^2^ and depended on the prevalence of obesity or GERD; the latter was chosen because it led to the highest sample size.

In the above equation, n = sample size, Z = Z statistic for a level of confidence (1.96 for 95% confidence level), P = expected prevalence or proportion of GERD that was 28.7% in Saudi Arabia based on on a previous study [8], and d = precision. The calculations indicated that 314 samples were needed to provide a 95% confidence interval (CI) and a precision of ±5%.

Data collection and questionnaire

In this study, all data were collected by researchers using an online questionnaire. The brief Arabic version of the World Health Organization Quality of Life questionnaire was used and was modified to match this study and its population; the questionnaire was validated by performing a pilot group test [20]. Cronbach’s alpha coefficient was used to check validity and determine the reliability and internal consistency of the questionnaire. Cronbach’s alpha pre- and post-test were 0.865 and 0.92, respectively, indicating very good reliability.

The questionnaire was distributed online among the general population in the Al-Baha region according to our criteria. All participants were informed in detail about the study aims and data confidentiality. Participants who agreed to take part in the study were asked to complete the questionnaire regarding sociodemographic data, symptoms and prevalence of GERD, exposed risk factors, and its impact on QOL through six domains: physical, psychological, level of dependence, social relationships, environmental, and overall QOL and general health perceptions. The GERD patients were determined by asking them if they had been diagnosed with GERD by a doctor.

Estimation of the QOL score

Regarding the QOL assessment, the questionnaire is formed of six domains: physical (four items), psychological (seven items), level of dependence (three items), social relationships (two items), environmental (eight items), and overall QOL and general health perceptions (two items). Each question is rated on a five-point Likert scale, in which five points mean the most satisfaction and one point means the lowest satisfaction. All items related to the same domains are added together to obtain the overall satisfaction of this domain.

Statistical analysis

The data were analyzed using the IBM SPSS Statistics for Windows, version 26.0 (released 2019, IBM Corp., Armonk, NY). All variables were summarized and reported across the study using descriptive statistics. The categorical variables were summarized and reported regarding the frequency distribution, and the chi-squared test was used to evaluate the associations. Logistic regression analysis was performed to identify the predictors of the dependent variable. A p-value of <0.05 was considered statistically significant.

## Results

Sociodemographic characteristics of the study population

A total of 720 patients completed the survey, of whom 406 were excluded because they were not obese. Therefore, 314 participants were included in the study according to the inclusion criteria. Of them, 132 (42%) were women, 133 (42.4%) were aged <30 years, and the mean ± standard deviation (SD) age of all the participants was 35.3 ± 12.9 years. The majority (311; 99%) of the population were Saudi, and 178 (56.7%) were married. A total of 181 (57.6%) were BMI obese grade I, 205 (65.3%) were non-smokers (only 40 (12.7%) were current smokers), and 120 (38.2%) were diagnosed with GERD, as presented in Table 1.

**Table 1:** Sociodemographic characteristics of the population of the study

Sociodemographic characteristics		n (%)
Age	Mean ± standard deviation	35.3 ± 12.9
	<30 years	133 (42.4)
	31–40 years	59 (18.8)
	41–50 years	84 (26.8)
	>50 years	38 (12)
Gender	Female	132 (42)
	Male	182 (58)
Body mass index	Obese I (30–34.9 kg/m^2^)	181 (57.6)
	Obese II (35–39.9 kg/m^2^)	95 (30)
	Obese III (≥40 kg/m^2^)	38 (12.4)
Nationality	Saudi	311 (99)
	Non-Saudi	3 (1)
Place of residence	Al-Baha	65 (20.7)
	Al-Aqiq	61 (19.4)
	Al-Makhwah	64 (20.4)
	Al-Mandaq	59 (18.8)
	Buljurashi	65 (20.7)
Marital status	Single	124 (39.5)
	Widow	3 (1)
	Married	178 (56.7)
	Divorced	9 (2.8)
Educational level	Elementary	10 (3.2)
	Middle school	12 (3.8)
	High school	92 (29.3)
	University	184 (58.6)
	Postgraduate	16 (5.1)
Occupation	Freelancer	12 (3.8)
	Student	51 (16.2)
	Government	147 (46.8)
	Private	29 (9.2)
	Unemployed	75 (24)
Income	<5,000 SAR	129 (41.1)
	5,000–10,000 SAR	91 (29)
	>10,000 SAR	94 (29.9)
Smoking status	Current	40 (12.7)
	Passive smoker	29 (9.3)
	Ex-smoker	40 (12.7)
	Non-smoker	205 (65.3)
Chronic diseases	Hypertension	30 (10)
	High cholesterol	54 (17)
	Heart diseases	3 (0.1)
	Diabetes	50 (16)
	Others	10 (3)
	No chronic diseases	194 (61.8)
Diagnosed with gastroesophageal reflux disease	Yes	120 (38.2)
	No	194 (61.8)
Had gastrointestinal tract surgery	Yes	34 (11)
	No	280 (89)

Prevalence of different symptoms of GERD among the participants

Regarding the frequency of the main symptoms of GERD, 66 (21%) did not have any symptoms of GERD, 63 in the non-GERD group and only three in the GERD group. A total of 123 (39.1%) had symptoms within the last year, 59 in the GERD group and 66 in the non-GERD group. Regarding the symptoms of GERD, 141 (44.9%) of the population had epigastric pain and a burning sensation more than once a week, 84 (70%) in the GERD group and 57 (29.4%) in the non-GERD group, and 126 (40.1%) did not have any epigastric pain, 28 (23.3%) in the GERD group and 98 (50.5%) in the non-GERD group. A total of 122 (38.9%) patients experienced food reflux more than twice a week, 82 (68.3%) in the GERD group and 40 (20.6%) in the non-GERD group. A total of 161 (51.3%) participants had heartburn more than twice a week, 84 (70%) in the GERD group and 77 (39.7%) in the non-GERD group. Only 93 (29.6%) of the patients did not have any heartburn symptoms, 25 (20.8%) in the GERD group and 68 (35.1%) in the non-GERD group, as presented in Table 2.

**Table 2:** Frequency of different symptoms of gastroesophageal reflux disease (GERD) among the population, n = 314

Symptoms	No symptom		Once a month		Every two weeks		More than once a week		More than once a day	
	GERD	Non-GERD	GERD	Non-GERD	GERD	Non-GERD	GERD	Non-GERD	GERD	Non-GERD
	n = 120	n = 194	n = 120	n = 194	n = 120	n = 194	n = 120	n = 194	n = 120	n = 194
	n (%)	n (%)	n (%)	n (%)	n (%)	n (%)	n (%)	n (%)	n (%)	n (%)
Epigastric pain or burning	28 (23.3%)	98 (50.5%)	1 (0.8%)	24 (12.4%)	7 (5.8%)	15 (7.7%)	32 (26.7%)	36 (18.6%)	52 (43.3%)	21 (10.8%)
Reflux of food into the throat	26 (21.7%)	108 (55.7%)	5 (4.2%)	18 (9.3%)	7 (5.8%)	28 (14.4%)	46 (38.3%)	28 (14.4%)	36 (30%)	12 (6.2%)
Heartburn	25 (20.8%)	68 (35.1%)	2 (1.7%)	23 (11.9%)	9 (7.5%)	26 (13.4%)	37 (30.8%)	45 (23.2%)	47 (39.2%)	32 (16.5%)
Chest pain	43 (35.8%)	132 (68%)	7 (5.8%)	20 (10.3%)	8 (6.7%)	7 (3.6%)	26 (21.7%)	18 (9.3%)	36 (30%)	17 (8.8%)
Chronic cough	44 (36.7%)	140 (72.2%)	5 (4.2%)	14 (7.2%)	4 (3.3%)	10 (5.2%)	33 (27.5%)	14 (7.2%)	34 (28.3%)	16 (8.2%)
Nausea and vomiting	45 (37.5%)	138 (71.1%)	6 (5%)	20 (10.3%)	10 (8.3%)	7 (3.6%)	27 (22.5%)	14 (7.2%)	32 (26.7%)	15 (7.7%)
Dysphagia	47 (39.2%)	150 (77.3%)	4 (3.3%)	10 (5.2%)	7 (5.8%)	8 (4.1%)	29 (24.2%)	12 (6.2%)	26 (24.2%)	33 (27.5%)
Sore throat	38 (31.7%)	128 (66%)	5 (4.2%)	20 (10.3%)	10 (8.3%)	11 (5.7%)	36 (30%)	23 (11.9%)	31 (25.8%)	12 (6.2%)
Dyspepsia	27 (22.5%)	115 (59.3%)	0	8 (4.1%)	9 (7.5%)	13 (6.7%)	42 (35%)	32 (16.5%)	42 (35%)	26 (13.4%)
Loss of appetite	39 (32.5%)	144 (74.2%)	6 (5%)	9 (4.6%)	6 (5%)	11 (5.7%)	28 (23.3%)	19 (9.8%)	41 (34.2%)	11 (5.7%)
Bloating	26 (21.7%)	94 (48.5%)	5 (4.2%)	12 (6.2%)	11 (9.2%)	10 (5.2%)	28 (23.3%)	43 (22.2%)	50 (41.7%)	35 (18%)

Overall duration of different symptoms of GERD among the population

Sixty-six patients showed no symptoms, three (2.5%) among the GERD group and 63 (32.5%) among the non-GERD group. Symptoms for <6 months were recorded among 16 (13.3%) of the GERD group and 31 (16%) of the non-GERD group. A symptoms duration of six months to one year was recorded among 43 (35.8%) of the GERD group and 35 (18%) of the non-GERD group, as presented in Table 3

**Table 3:** Overall duration of different symptoms of gastroesophageal reflux disease (GERD) among the population

For how long did you feel these symptoms?	GERD (n = 120)	Non-GERD (n = 194)
	n (%)	n (%)
I don’t have symptoms	3 (2.5)	63 (32.5)
<6 months	16 (13.3)	31 (16)
6 months–1 year	43 (35.8)	35 (18)
1–3 years	32 (26.7)	36 (18.6)
>3 years	26 (21.7)	29 (14.9)

Food habits, risk, and relieving factors associated with GERD

Regarding habits and aggravating factors, only 151 (48%) of patients avoided spicy food to relieve symptoms, 113 (94.2%) in the GERD group and 38 (19.6%) in the non-GERD group. A total of 100 (68.7%) of the population avoid fast food, 54 (45%) in the GERD group and 46 (23.7%) in the non-GERD group. Regarding risk factors of GERD, 57 (47.5%) of GERD patients ate large amounts of food and 36 (30%) of them ate spicy food.

Regarding drugs used that aggravated symptoms, only 51 (16.2%) of the GERD and non-GERD had used non-steroidal anti-inflammatory drugs (NSAIDs), 20 (16.7%) and 31 (16%), respectively. Regarding the main drug used to reduce GERD symptoms, results showed 98 (31.2%) of patients used omeprazole, 42 (35%) of the GERD group and 56 (28.9%) of the non-GERD group, as presented in Table 4.

**Table 4:** Risk factors, relieving factors, and habits among the population of the study, n = 314

Risk factors, relieving factors, and habits		GERD (n = 120)	Non-GERD (n = 194)
		n (%)	n (%)
Food you are avoiding to reduce your symptoms	Spicy food	113 (94.2)	38 (19.6)
	Fast food	54 (45)	46 (23.7)
	Fatty foods	56 (46.6)	102 (52.6)
	I don’t avoid	15 (12.5)	72 (37)
Risk factors that apply to you	Eating large amounts of food	57 (47.5)	97 (50)
	Lack of physical activity	65 (54)	108 (55.7)
	Eating acidic foods or drinks	43 (35.8)	38 (19.6)
	Eating spicy food	36 (30)	117 (60.3)
	Drink soft drinks	59 (49)	54 (27.8)
	Family history	17 (14.2)	8 (4.1)
	Any of these factors	13 (10.8)	24 (12.4)
Are you using one of these medications?	NSAID (aspirin/brufen)	20 (16.7)	31 (16)
	Proton pump inhibitor (PPI)	30 (25)	40 (20.6)
	Antidepressant	9 (7.5)	9 (4.6)
	Anti-anxiety	2 (1.6)	2 (1)
	Nothing	58 (48.3)	113 (58.2)
Did you take medication to reduce the symptoms of GERD?	Always	20 (16.7)	23 (11.8)
	Usually	81 (15)	31 (16)
	Sometimes	31 (25.8)	51 (26.4)
	Never	51 (42.5)	89 (45.8)
Drug dose you use	Controloc	8 (6.7)	14 (7)
	Risek	15 (12.5)	17 (8.7)
	Omeprazole	42 (35)	56 (28.9)
	Others	6 (5)	18 (9.3)

Association between GERD and sociodemographic characteristics

The chi-squared test was applied to test the association between different sociodemographic characteristics and GERD. The results showed a statistically significant relationship between GERD and gender (p = 0.014). In addition, there was a statistically significant relationship between GERD and smoking status (p = 0.004). Furthermore, a statistically significant relation with place of residence, educational level, occupation, and income (the p-values were 0.001, 0.005, 0.04, and 0.03, respectively), as shown in Table 5.

**Table 5:** Association between gastroesophageal reflux disease (GERD) and sociodemographic characteristics, n = 314 ‎A p-value of <0.05 is considered statistically significant.

Characteristics		GERD (n = 120)	Non-GERD (n = 194)	p-value
		n (%)	n (%)	
Gender	Female	40 (33.3)	92 (47.4)	0.014
	Male	80 (66.7)	102 (52.6)	
Age	<30 years	50 (41.7)	83 (42.8)	0.43
	31–40 years	22 (18.3)	37 (19.1)	
	41–50 years	29 (24.2)	55 (28.3)	
	>50 years	19 (15.8)	19 (9.8)	
BMI	Obese I (30–34.9 kg/m^2^)	65 (54.2)	116 (59.8)	0.5
	Obese II (35–39.9 kg/m^2^)	38 (31.6)	57 (29.4)	
	Obese III (≥40 kg/m^2^)	17 (14.1)	21 (10.8)	
Nationality	Saudi	120 (100)	191 (98.5)	0.17
	Non-Saudi	0	3 (1.5)	
Place of residence	Al-Baha	5 (4.2)	60 (30.9)	0.001
	Al-Aqiq	12 (10)	49 (25.3)	
	Al-Makhwah	25 (20.8)	39 (20.1)	
	Al-Mandaq	37 (30.8)	22 (11.3)	
	Buljurashi	41 (34.2)	24 (12.4)	
Marital status	Single	49 (40.8)	75 (38.7)	0.56
	Widow	0	3 (1.5)	
	Married	68 (56.7)	110 (56.7)	
	Divorced	3 (2.5)	6 (3.1)	
Educational level	Elementary	6 (5)	4 (2)	0.005
	Middle school	9 (7.5)	3 (1.5)	
	High school	39 (32.5)	53 (27.4)	
	University	64 (53.3)	120 (61.9)	
	Postgraduate.	2 (1.7)	14 (7.2)	
Occupation	Freelancer	7 (5.8)	5 (2.6)	0.04
	Student	12 (10)	39 (20.1)	
	Government	53 (44.2)	94 (48.5)	
	Private	13 (10.8)	16 (8.2)	
	Unemployed	35 (29.2)	40 (20.6)	
Income	<5,000 SAR	42 (35)	87 (44.9)	0.03
	5,000–10,000 SAR	45 (37.5)	46 (23.7)	
	>10,000 SAR	33 (27.5)	61 (31.5)	
Smoking status	Current	22 (18.3)	18 (9.3)	0.004
	Passive smoker	8 (6.7)	21 (10.8)	
	Ex-smoker	22 (18.3)	18 (9.3)	
	Non-smoker	68 (56.7)	137 (70.6)	

QOL domains among the population

The mean and SD of the physical domain were higher in the non-GERD participants than in the GERD participants (12.8 ± 3.4 vs. 11.3 ± 2.6, respectively), and on applying the chi-squared test, the results showed there was a significant association (p = 0.001). The psychological domain showed a non-significant result among both groups (p = 0.152).

The social and environmental domains also showed a statistically significant difference between both groups (p = 0.001 and 0.017, respectively). The overall QOL was higher in the non-GERD patients than in the GERD patients (p = 0.001), as shown in Table 6.

**Table 6:** Comparisons of different domains of quality of life (QOL) and sociodemographic characteristics of the population, n = 314 ‎A p-value of <0.05 is considered statistically significant.

Domains of QOL	GERD (n = 120)	Non-GERD (n = 194)	p-value
	Mean ± SD	Mean ± SD	
Physical (4 items)	11.3 ± 2.6	12.8 ± 3.4	0.001
Psychological (7 items)	19.1 ± 5.2	21.5 ± 5.8	0.152
Level of independence (3 items)	8.5 ± 2	9.7 ± 2.5	0.002
Social relationships (2 items)	5.5 ± 2.3	6.7 ± 2.3	0.0001
Environment (8 items)	22 ± 6.9	25.8 ± 7.5	0.017
Overall quality of life and general health perception (2 items)	5.5 ± 1.8	6.7 ± 2.2	0.001

Logistic regression analysis of the associated sociodemographic variables

Logistic regression analysis was performed to identify the predictors that affect the incidence of GERD. The results showed that the odds of having GERD were 1.8 times greater among men than among women (p = 0.014, which is statistically significant). The age group of <30 years was the reference, and the odds were higher in the group of those aged >50 years (odds ratio (OR) = 1.7), with a statistically insignificant value.

Regarding smoking status, the non-smokers were the reference group and the odds of having GERD in current smokers were 2.6 times the odds of having GERD among non-smokers and 2.5 times greater than passive smokers (p = 0.003 and 0.009, respectively). Regarding obesity, the results showed that grade I obesity had a lower risk of GERD development than grades II and III, and the results were statistically insignificant.

Place of residence showed a significant result as Al-Baha residents had odds of having GERD less than in the other towns. Buljurashi participants had a 20.5 times higher risk of developing GERD than Al-Baha people (p = 0.001). Furthermore, there was a significant difference in Al-Mandaq and Al-Makhwah in comparison with Al-Baha (p = 0.001 for each).

Educational level has a significant difference. The odds of having GERD in patients with elementary schooling were 10 times those in postgraduate patients. Regarding hypertension, the odds of being diagnosed with GERD in patients with hypertension were 2.6 times higher than in patients who did not have hypertension (p = 0.007).

In addition, cholesterol levels showed a significant result, as the odds of having GERD in patients with high levels were 2.4 times those among patients who did not have high cholesterol levels (p = 0.005). Other predictors are shown in Table 7.

**Table 7:** Univariate and multivariate binary logistic regression analysis of factors associated with the incidence of gastroesophageal reflux disease among the study population, n = 314 ‎A p-value of <0.05 is considered statistically significant.

Variable	Odds ratio	Confidence interval	p-value
Sex			
Male	1.8	1.1–2.9	0.014
Female	Reference		-
Age group			
<30 years	Reference	-	-
31–40 years	1	0.5–1.9	0.9
41–50 years	0.8	0.5–1.5	0.6
>50 years	1.7	0.8–3.5	0.13
Smoking state			
Current	2.6	1.4–5	0.003
Passive smoker	2.5	1.3–4.8	0.009
Ex-smoker	0.7	0.3–1.8	0.559
Non-smoker	Reference	-	-
Obesity			
Obese I (30–34.9 kg/m^2^)	Reference	-	-
Obese II (35–39.9 kg/m^2^)	1.2	0.7–2	0.5
Obese III (≥40 kg/m^2^)	1.5	0.7–3	0.3
Income			
<5,000 SAR	0.8	0.27–2.4	0.7
5,000–10,000 SAR	1.8	0.7–3.2	0.27
>10,000 SAR	Reference	-	-
Place of residence			
Al-Baha	Reference	-	-
Al-Aqiq	3	1–8.9	0.057
Al-Makhwah	7.7	2.7–21.8	0.001
Al-Mandaq	20.2	7–57.9	0.001
Buljurashi	20.5	7.2–58	0.001
Marital status			
Single	2.3	0.4–12.5	0.3
Married	1.4	0.3–0.6	0.7
Divorced	Reference	-	-
Educational level			
Elementary	10.5	1.5–73.7	0.01
Middle school	5.1	1.1–24	0.03
High school	21	2.9–151	0.003
University	3.7	0.8–17	0.08
Postgraduate	Reference	-	-
Occupation			
Freelancer	2.5	0.7–8	0.13
Student	0.5	0.3–1.1	0.1
Government sector	Reference	-	-
Private sector	1.4	0.6–3.2	0.37
Unemployed	1.5	0.8–2.7	0.12
Chronic diseases			
Hypertension			
Yes	2.6	1.3–5.3	0.007
No	Reference	-	
High cholesterol			
Yes	2.4	1.3–4.5	0.005
No	Reference	-	
Diabetes			
Yes	1.5	0.8–2.9	0.2
No	Reference	-	

## Discussion

The present cross-sectional study was carried out to estimate the prevalence of GERD, its different symptoms, the feeding habits of patients, its associated risk factors, and its effects on the QOL among the population of Al-Baha region, Saudi Arabia. This study showed a relatively high prevalence of GERD (120 out of 314, or 38.2%). This result is low if we compare it with the study conducted by Eslami et al. [21] in Iran among obese patients, as the prevalence was 56% (285 out of 505). The present study results were high if compared with the study of Alsuwata et al. [22], which was conducted in the overall areas of Saudi Arabia and found that 28.7% of the population had GERD. In another study conducted in Riyadh, 34.1% of the participants were obese, and the prevalence of GERD was found to be 45.4% [8]. Kariri et al. reported that 32.2% of southwestern (Jazan region), Saudi Arabia, have GERD, which is lower compared with the results of our study [23]. A systematic review conducted in 2020 reported that the prevalence of GERD is 19.5% in North America, 12.8% in Latin America, 12.9% in Asia, 14% in Europe, and 8.7-33.1% in the Middle East [24]. This high discrepancy in prevalence is thought to be due to different assessment tools and scales with different feeding habits, lifestyles, and races.

Regarding gender, 66.7% of the patients were male, and the difference was statistically significant. After conducting logistic regression, men were 1.8 times more likely than women to develop GERD (OR: 1.8; 95% CI 1.1-2.9), which was similar to a previous systematic review in 2016, which reported that men are more likely to develop GERD than women [25]. Some other previous studies did not find any association between GERD and gender [8,9]. This may be explained by the effect of the estrogen hormone, which has an anti-inflammatory action and protects females from acid reflux and subsequently erosive esophagitis and GERD [26,27].

The American College of Gastroenterology has stated that obesity is considered one of the major risk factors for GERD and recommends losing weight as a lifestyle modification to relieve its symptoms. However, in the present study, no significant association was found between BMI and GERD, although many studies had reported an association between higher BMI levels and GERD [28,29]. Regarding the main symptoms of GERD, our study found that epigastric pain and burning sensation are the most common gastrointestinal manifestations affecting GERD patients, representing 70%, followed by food reflux at 68%; this is very high in comparison with other studies, which reported approximately 49% and 47.5% for heartburn and food reflux, respectively [22,30]. Another study conducted in the USA found that heartburn is the most common symptom, and it affects only 45% of obese patients with a BMI >35 kg/m^2^ and 56% of obese patients with a BMI ≤35 kg/m^2^ [31]. Almadi et al. [8] conducted a study in Saudi Arabia and found that heartburn represented the main complaint in 38% of patients; food regurgitation represented 35% of patients, but only 34.1% of the participants were obese.

Smoking is considered a main risk factor for GERD, which impairs the LES and leads to acid reflux from the stomach to the lower esophagus, causing GERD symptoms [32-34]. In the present study, 12.7% were current smokers, 12.7% were ex-smokers, and 9.3% were passive smokers. We found a statistically significant association between smoking and GERD (p = 0.004), and on conducting logistic regression analysis, current smokers were 2.6 times more likely than non-smokers to develop GERD (OR = 2.6; 95% CI 1.4-5; p = 0.003), while passive smokers were 2.5 times more likely to get GERD (OR =2.5; 95% CI 1.3-4.8; p = 0.009). Similar findings were reported by Kariri et al. [23] in their study of Jazan populations, who found a strong association between smoking and GERD (p < 0.01), and Awadalla et al. [35] in their study on King Khalid University students, who found current smokers were 1.71 times more prone to GERD than non-smokers (OR = 1.71); however, Atta et al. [36], who conducted their study on Jeddah Medical students, did not find any association. Therefore, cessation of smoking is one of the most important lines of research for GERD control.

Dietary habits are major risk factors for GERD exacerbation. Large meals, spicy food, high-fat meals, lack of physical activity, and NSAID abuse are all associated with increasing gastric acid production, impairing LES, and refluxing food into the esophagus. A systematic review conducted in 2021 reported that these dietary habits and lifestyles are positively correlated with GERD [37]. In the present study, the prevalence of bulimic eating among GERD patients was 54%, eating spicy food 30%, lack of physical activity 54%, taking NSAIDs 16.7%, and acidic food and drinks 35.8%.

There are common risk factors for GERD and hypertension, such as obesity, age, male gender, and smoking [38-40]. Suyu et al. [41] found that hypertension is a positive predictor for GERD, especially silent GERD. In the present study, hypertensive patients were 2.6 times more liable than non-hypertensive patients to get GERD (OR = 2.6; 95% CI 1.3-5.3; p = 0.007). Furthermore, hyperlipidemia is associated with GERD and represents a major risk factor, and the current study found that hyperlipidemic patients have 2.4 more risk of developing GERD (OR = 2.4; 95% CI 1.3-4.5; p = 0.005) [38].

On assessing the QOL of the study participants, we found that the non-GERD group had a significantly higher QOL regarding the physical, social, level of independence, overall QOL, and health perception; all showed a strong association with GERD (p < 0.05). These results were similar to many previous studies conducted in Saudi Arabia, which stated that GERD affects the physical state and daily activities of the patients [42]. In Turkey, a study published by Mungan [43] reported that GERD patients have a significantly lower QOL compared with the general population. Alshammari et al. and Meyiz et al. showed similar results [44,45].

The study has some limitations. Cross-sectional studies cannot generalize the results to the whole population and cannot determine causality. Moreover, patients assessed their own symptoms, so this may have some recall bias. No specific criteria were used to diagnose patients with GERD; they were considered as having GERD according to their answer to the question of whether they had been diagnosed with GERD by a doctor. No control group was used to compare with the study group. The QOL questionnaire was self-reported by participants; therefore, a different understanding is possible according to age and level of education, which may present a selection bias.

## Conclusions

Based on the results of the present study, the prevalence of GERD among obese patients in the Al-Baha region is high and has a bad impact on their QOL. We proved that male gender, smoking, hyperlipidemia, and hypertension are statistically significant risk factors that increase the incidence of GERD among Al-Baha region residents. Therefore, avoiding smoking, spicy food, abusing NSAIDs, and eating too much food without regular physical activity are important protective measures for GERD. Our study helped fill the gap related to this topic in the Al-Baha region. Public health awareness campaigns and health programs are needed to increase the awareness of the population about these risk factors, bad lifestyles, and triggering habits that exacerbate symptoms of GERD to improve QOL and prevent complications.
